# New machine learning method for image-based diagnosis of COVID-19

**DOI:** 10.1371/journal.pone.0235187

**Published:** 2020-06-26

**Authors:** Mohamed Abd Elaziz, Khalid M. Hosny, Ahmad Salah, Mohamed M. Darwish, Songfeng Lu, Ahmed T. Sahlol

**Affiliations:** 1 Faculty of Science, Zagazig University, Zagazig, Egypt; 2 School of Cyber Science and Engineering, Huazhong University of Science and Technology, Wuhan, China; 3 Faculty of Computers and Informatics, Zagazig University, Zagazig, Egypt; 4 Faculty of Science, Assiut University, Assiut, Egypt; 5 Faculty of Specific Education, Damietta University, Damietta, Egypt; Politechnika Slaska, POLAND

## Abstract

COVID-19 is a worldwide epidemic, as announced by the World Health Organization (WHO) in March 2020. Machine learning (ML) methods can play vital roles in identifying COVID-19 patients by visually analyzing their chest x-ray images. In this paper, a new ML-method proposed to classify the chest x-ray images into two classes, COVID-19 patient or non-COVID-19 person. The features extracted from the chest x-ray images using new Fractional Multichannel Exponent Moments (FrMEMs). A parallel multi-core computational framework utilized to accelerate the computational process. Then, a modified Manta-Ray Foraging Optimization based on differential evolution used to select the most significant features. The proposed method evaluated using two COVID-19 x-ray datasets. The proposed method achieved accuracy rates of 96.09% and 98.09% for the first and second datasets, respectively.

## 1. Introduction

COVID-19 is a global challenge that should be addressed by all scientific means. Medical image analysis is a well-known approach that could be beneficial in the diagnosis of COVID-19. Severe Acute Respiratory Syndrome (SARS) and COVID-19 belong to the same family of Coronaviruses, where the detection of SARS cases using chest images proposed by several methods [1–3] and for pneumonia detection in general [4].

ML has demonstrated high performance for several image processing applications such as image analysis [5, 6], image classification [7], and image segmentation [8]. Image classification achieved by extracting the import features from the images by a descriptor (e.g., SIFT [9] and image moment [10]), and then these features can be used in the classification task using classifiers such as SVM [11]. In contrast to handcrafted features, deep neural network-based methods [12] provides high performance in classifying the images according to the extracted features. According to the characteristics of ML, several efforts utilized machine learning-based methods to classify the chest x-ray images into COVID-19 patient class or normal case class. All of these efforts utilized deep learning-based approaches. For instance, the authors proposed a CNN model for the automatic diagnosis of COVID-19 from chest x-ray images [13]. Their reported classification accuracy is 96.78% using MobileNet architecture [13]. Similarly, the conducted research in [14] utilized the transfer learning approach. The reported accuracy rate is 97% and 87% accuracy for InceptionV3 and 87% for Inception-ResNetV2, respectively.

Recently, orthogonal moments and their variants are powerful tools used in many image processing and pattern recognition applications. Feature extraction using the image moments successfully reported for several applications [15] and [16]. For instance, combining orthogonal quaternion Polar Harmonic Transform moments with optimization algorithms for image representation and feature selection has been successfully reported in color galaxies images classification [17].

The motivation of this research is to propose an accurate classification method for COVID-19 chest x-ray image depends on combining the strength of two techniques. First, a new image descriptor, FrMEMs. Second, a modified feature selection technique based on Manta-Ray Foraging Optimization and differential evolution (MRFODE).

In this work, we proposed a method of COVID-19 chest x-ray image classification. The proposed method extracts the features from chest x-ray images using FrMEMs moment. Then the extracted features are divided into testing and training sets. Followed by using the MRFODE algorithm to reduce these features an d remove the redundant and irrelevant features. This process achieved by generating a set of solutions and computing the fitness value for each of them using the KNN classifier based on a training set with determining the best of them. Then applying the operators of MRFO in the exploration phase; however, in the exploitation phase, the probability of each solution is computed using its fitness value. According to specified criteria, the solution updated either using DE or the operators of MRFO. The process of updating solutions stopped when reached to terminal conditions. The best solution used to remove the irrelevant features from the testing set and compute the label of the COVID-19 image dataset.

The main contributions of this study are:

1. Proposed a COVID-19 classification method depends on the properties of orthogonal moment features and feature selection techniques.
2. Deriving a new set of descriptors, FrMEMs, to extract the features from the COVID-19 images.
3. Developed a new feature selection method based on improving the behavior of Manta Ray Foraging Optimization (MRFO) using Differential evolution (DE).
4. Evaluate the performance of the proposed model using two COVID-19 x-ray datasets.
5. Compare the results with other feature selection methods and DNN techniques.

The organization of this paper is as follows. In Section 2, the proposed model utilized FrMEMs and the bio-inspired optimization algorithm represented. The experimental results of the proposed model discussed in Section 3. Finally, the paper concluded in Section 4.

## 2. Proposed image-based classification method

Image moments defined as projections of image functions onto a polynomial basis where the image moments used to extract global and local features from these images [18]. Generally, projection of digital images using orthogonal polynomials with fractional orders results in orthogonal moments of fractional orders which able to extract both coarse and fine features from the input digital images. In this paper, new orthogonal Exponent moments of fractional-orders derived. Then, these moments utilized to extract high accurate 961 features from each COVID-19 input image. The intrinsic properties of the new image moments are:

1. These orthogonal moments are successfully able to represent digital images for low and high orders.
2. The orthogonal moments are invariants to geometric transformations, which is an essential property for classification and recognition applications.
3. The orthogonal moments are robust to noise.
4. Fast and inexpensive computation requirements make them favorable for real-time applications.

### 2.1. Feature extraction

A few years ago, Hu et al. [19] defined the orthogonal exponent moments as:

$$FrM_{pq}=\frac{1}{4\pi }\int_{0}^{2\pi }\int_{0}^{1}f\left(r,\theta \right){\left[E_{pq}\left(r,\theta \right)\right]}^{*}rdrd\theta$$ (1)

where the order, p, and the repetition, q, are 0,±1,±2,±3,… ….; $\hat{i}=\sqrt{-1};\;{\left[∙\right]}^{*}$ refers to the complex conjugate process; *E*_*pq*_(*r*,*θ*) refers to the exponent basis functions which defined as:

$$E_{pq}\left(r,\theta \right)=T_{p}\left(r\right)e^{-\hat{i}q\theta },$$ (2)

with

$$T_{p}\left(r\right)=\sqrt{\frac{2}{r}}\;e^{-\hat{i}2\;\pi p\;r}$$ (3)

We generalized *E*_*pq*_(*r*,*θ*) of integer orders in the domain [0,1]×[0,2*π*] and converted to the fractional-order form, $W_{pq}^{\alpha }(r,\theta )$, with a real-values parameter $\alpha \;\epsilon \;R^{+}$ in the same domain as follows:

$$W_{pq}^{\alpha }\left(r,\theta \right)=T_{p}\left(r,\alpha \right)\;e^{-\hat{i}q\theta }$$ (4)

where

$$T_{p}\left(r,\alpha \right)=r^{\alpha -1}\sqrt{\frac{2}{r^{\alpha }}}\;e^{-\hat{i}2\pi pr^{\alpha }}$$ (5)

The basis functions of fractional-order, $W_{pq}^{\alpha }(r,\theta )$, are orthogonal where:

$$\int_{0}^{2\pi }\int_{0}^{1}W_{pm}^{\alpha }\left(r,\theta \right){\left[W_{qn}^{\alpha }\left(r,\theta \right)\right]}^{*}rdrd\theta =\frac{4\pi }{\alpha }\delta_{pm}\delta_{qn}$$ (6)

In this paper, the authors utilized the multi-channel approach [20, 21] in which the input color images processed using the RGB color model where the *R*−, *G*− & *B*−channels are expressed using *f*_*R*_(*r*,*θ*),*f*_*G*_(*r*,*θ*) & *f*_*B*_(*r*,*θ*) respectively. The multi-channel orthogonal fractional-order exponent moments are:

$$FrM_{pq}=\frac{\alpha }{4\pi }\int_{0}^{2\pi }\int_{0}^{1}f_{c}\left(r,\theta \right)\;r^{\alpha -1}\sqrt{\frac{2}{r^{\alpha }}}\;e^{-\hat{i}2\pi pr^{\alpha }}e^{-iq\theta }rdrd\theta$$ (7)

Assume the rotation of the original image, *f*_*c*_(*r*,*θ*), with an angle *β*, then the rotated image, $f_{C}^{\beta }(r,\theta )$, is:

$$f_{C}^{\beta }\left(r,\theta \right)=f_{c}\left(r,\theta -\beta \right)$$ (8)

Let $\hat{\theta }=\theta -\beta$, then $\theta =\hat{\theta }+\beta$ and $d\theta =d\hat{\theta }$, then using the Eq (8) in (7) yields:

$$FrM_{pq}\left(f_{C}^{\beta }\left(r,\theta \right)\right)=FrM_{pq}\left(f_{c}(r,\theta )\right)e^{iq\beta }$$ (9)

Based on the properties of Euler function, |e^iqβ^| = 1, So, equation (E10) is simplified as:

$$|FrM_{pq}(f_{C}^{\beta }\left(r,\theta \right))\left|=|FrM_{pq}(f_{c}\left(r,\theta \right))\right|$$ (10)

This equation proves that the magnitude values of FrMEMs are unchanged with any rotation in the input image.

Wang et al. [22] showed that circular orthogonal moments achieved the scaling invariance when the input color images mapped into the unit circle. In this work, the input images interpolated to fit the unit-circle domain. Thus, the computed FrMEMs are scaling invariants. The central FrMEMs, are derived in a similar way to [23]. The FrMEMs calculated with high accuracy using the kernel-based approach [24, 25].

### 2.2. Parallel implementation

The parallel implementation is a recent trend used to accelerate the intensive computing of image moments, especially for large-sized images and high moment orders. The emergence of new parallel architectures enriches the efforts toward this goal. Qin et al. [26] proposed a parallel recurrence method to accelerate the implementation of the Zernike moment. In this context, Deng et al. [27] extended the work of Qin and his colleague. They implemented a parallel-friendly method for moment computation and image reconstruction based on Zernike moment. Recently, Salah et al. [28] proposed a parallel computational method to accelerate the computational process of the polar harmonic transforms of integer-orders.

In the same direction, we proposed a parallel implementation of the FrMEMs on multi-core CPU architecture. This parallel implementation provided to cope with the increasing size of the chest x-ray dataset. The FrMEMs consists of (*pmax*+1)×(*qmax*+1) moment component. These moment components computations are independent. Each moment component has a unique combination of *p* and *q* values. For instance, for a fractional moment order of 5, there are 36 separate moment components.

**Fig 1:**
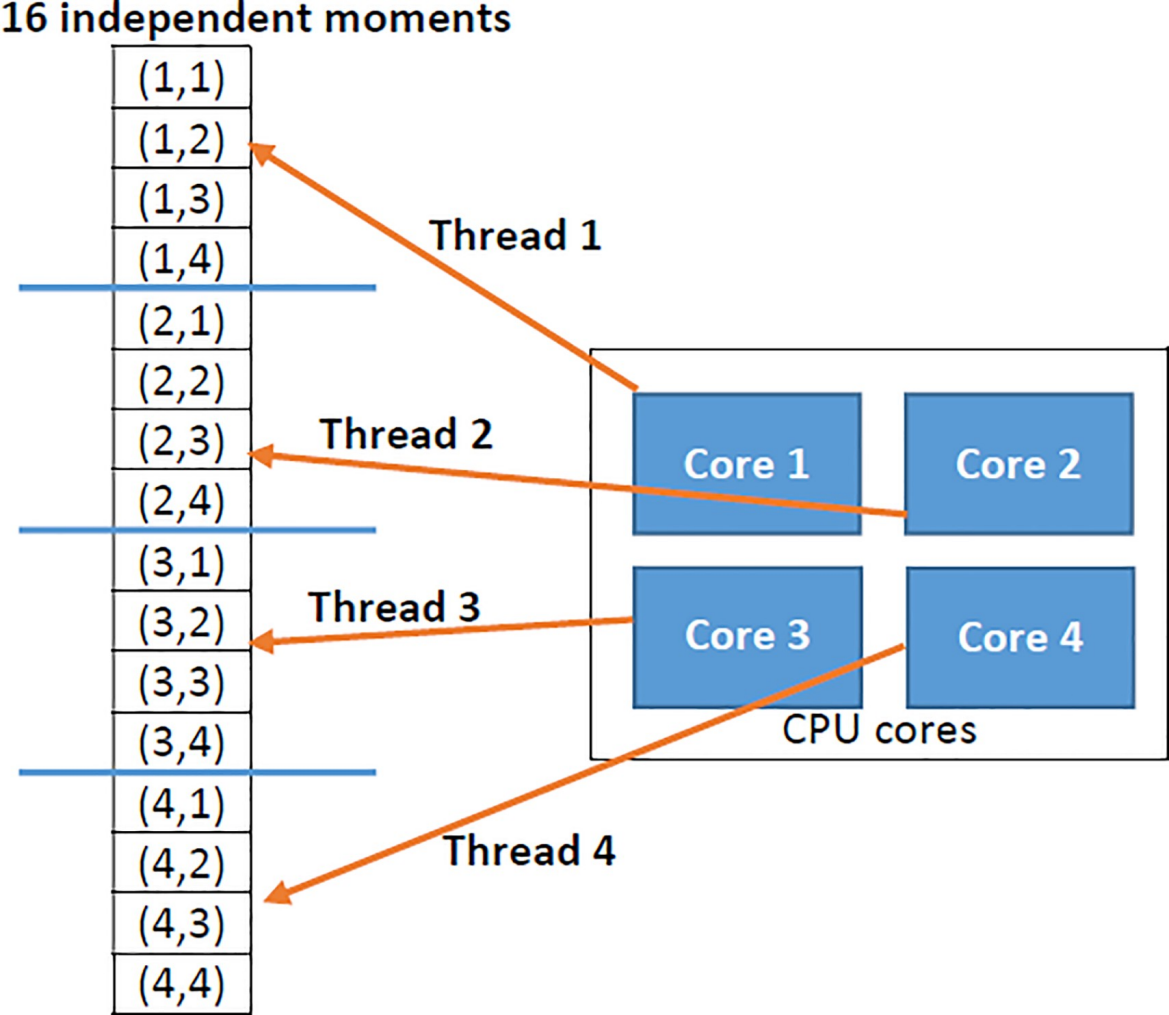
Parallel implementation of FrMEMs moment on 4-cores CPU.

In Fig 1, the proposed parallel implementation of FrMEMs moment depicted. The multi-core CPU has four cores; each core computes a portion of the moment components.

### 2.3. Feature selection

In this part, we introduced the modified Manta-Ray Foraging Optimization (MRFO) based on Differential Evolution (DE) as a feature selection method. However, the basics of MRFO and DE discussed firstly.

#### 2.3.1. Manta Ray Foraging Optimization *(MRFO)*

In general, the MRFO simulates the behaviors of three foragings, including cyclone foraging, Chain foraging, and somersault foraging [29]. The details of each foraging given in the following subsections.

*Chain foraging*. In the MRFO, the foraging chain formed by using the manta rays' line up head-to-tail. Besides, the movement of each agent, except the first one, is in the direction of the food and the agent in front of it which means the current agent (*x*_*i*_(*t*),*i* = 1,2…,*N*) at iteration (*t*) is updated depends on the position of best agent and the agent in front of it. This process formulated as in the following equation:

$$x_{i}^{d}\left(t+1\right)=\{\begin{array}{c} x_{i}^{d}\left(t\right)+r\times \left(x_{best}^{d}\left(t\right)-x_{i}^{d}\left(t\right)\right)+\alpha \times \left(x_{best}^{d}\left(t\right)-x_{i}^{d}\left(t\right)\right),\;i=1\\ x_{i}^{d}\left(t\right)+r\times \left(x_{i-1}^{d}\left(t\right)-x_{i}^{d}\left(t\right)\right)+\alpha \times \left(x_{best}^{d}\left(t\right)-x_{i}^{d}\left(t\right)\right)\;otherwise \end{array}$$ (11)

where *r*∈[0,1] refers to random vector and $x_{best}^{d}(t)$ represents the best agent (in MRFO refers to the plankton with high concentration) at *d*-th dimension. *α* is a weight coefficient, and defined as:

$$\alpha =2\times r\times \sqrt{\left|\log\left(r\right)\right|}$$ (12)

*Cyclone foragin*. In this foraging, the manta rays will construct a long chain foraging, and they are swimming towards the source of the food in a spiral movement. This process means that each agent will follow the front agent, and its movement is in the direction of the best solution along the spiral. Therefore, the updating process of the current agent formulated as:

$$x_{i}^{d}\left(t+1\right)=\{\begin{array}{c} x_{best}^{d}\left(t\right)+r\times \left(x_{best}^{d}\left(t\right)-x_{i}^{d}\left(t\right)\right)+\beta \times \left(x_{best}^{d}\left(t\right)-x_{i}^{d}\left(t\right)\right)\;i=1\\ x_{best}^{d}\left(t\right)+r\times \left(x_{i-1}^{d}\left(t\right)-x_{i}^{d}\left(t\right)\right)+\beta \times \left(x_{best}^{d}\left(t\right)-x_{i}^{d}\left(t\right)\right)\;i=2,\ldots ,N \end{array}$$ (13)

$$\beta =2e^{r_{1}\frac{T-t+1}{T}}\times \sin\left(2\pi r_{1}\right)$$ (14)

where *r*_1_∈[0,1] is a random number, *T* is the total number of generations.

Followed [29], the agents forced to find a new position far from $x_{best}^{d}(t)$ by using a random number as reference to them in the search space instead of the best agent. This can be formulated as:

$$x_{i}^{d}\left(t+1\right)=\{\begin{array}{c} x_{rand}^{d}\left(t\right)+r\times \left(x_{rand}^{d}\left(t\right)-x_{i}^{d}\left(t\right)\right)+\beta \times \left(x_{rand}^{d}\left(t\right)-x_{i}^{d}\left(t\right)\right)\;i=1\\ x_{rand}^{d}\left(t\right)+r\times \left(x_{i-1}^{d}\left(t\right)-x_{i}^{d}\left(t\right)\right)+\beta \times \left(x_{rand}^{d}\left(t\right)-x_{i}^{d}\left(t\right)\right)\;otherwise \end{array}$$ (15)

where $x_{rand}^{d}(t)$ is a random agent generated in the search space using the following equation:

$$x_{rand}^{d}(t)=LB^{d}+rand\times \left(UB^{d}-LB^{d}\right)$$ (16)

*Somersault foraging*.

In this foraging, each agent swims to and around the position of the food (is called pivot). Thus, the agents update their positions using the following equation:

$$x_{i}^{d}\left(t+1\right)=x_{i}^{d}\left(t\right)+S\times \left(r_{2}\times x_{best}^{d}\left(t\right)-r_{3}\times x_{i}^{d}\left(t\right)\right),i=1,2,\ldots ,N$$ (17)

where ***r***_**2**_ and *r*_3_ are random numbers belong to [0,1].

#### 2.3.2. Differential evolution

In this section, the mathematical modeling of Differential evolution (DE) introduced one of the most popular [30]. The DE, similar to other MHs, begins by setting the initial value for a set of agents *X*, then calculate the fitness value for each agent. Thereafter, mutation operator is applied to *X*_*i*_ and it is formulated as.

$$Z_{i}=X_{r1}+F\times \left(X_{r2}-X_{r3}\right)$$ (18)

In Eq (18), *r*1,*r*2, and *r*3 refer to random indices, but they are different from *i*. *F* represents the mutation scaling factor. The next step is to apply the crossover operator to generate a new agent, and defined as:

$$V_{i}=\{\begin{array}{c} Z_{i}\;r<C_{r}\\ X_{i}\;otherwise \end{array}$$ (19)

In Eq (19), *C*_*r*_ is the probability of the crossover, and *r*∈[0,1] is a random value. The next process is to compute the fitness value of *V*_*i*_ and compared it with *f*(*X*_*i*_) to update the value of the current agent *X*_*i*_ as in the following equation:

$$X_{i}=\{\begin{array}{c} V_{i}\;f\left(V_{i}\right)<f\left(X_{i}\right)\\ X_{i}\;otherwise \end{array}$$ (20)

#### 2.3.3. Enhanced MRFO based on DE as feature selection

In this section, the developed COVID-19 x-ray image classification model based on the extracted features using the FrMEMs and implemented an enhanced version from the MRFO based on DE, which called MRFODE presented. The developed method begins by extracting the features from the input images, either COVID-19 or Non-COVID-19, using FrMEMs. Then MRFODE generates a set of *N* agents; each of them is a solution for the FS problem (i.e., a subset of selected features). After that, the fitness value for each agent is computed, which indicates the quality of the selected features corresponding to the ones in the Boolean version of each agent. The best agent that has the best fitness value is determined and used in updating the position of agents using the operators of the traditional MRFO. Then, the terminal condition (if they reached) checked. Finally, they stop updating or repeat the process. The main steps of the proposed COVID-19 image classification contain three phases where the details of each stage discussed in a separate subsection.

In the first phase, the input x-ray images received then FrMEMs applied to extract a set of features (*D*_*Feat*_) from these images. The extracted features split into two, training and testing sets, which represent 80% and 20% respectively from the total number of images.

The second phase begins by setting a random value for a set of N agents using Eq (21).

$$x_{i}=LB_{i}+rand\times \left(UB_{i}-LB_{i}\right)$$ (21)

Each agent is converted to binary using the following equation:

$$x_{i}=\{\begin{array}{c} 1,\;if\frac{1}{1+e^{-X_{i}}}>0.5\\ 0,\;otherwise \end{array}$$ (22)

This sigmoid function is applied since it provides high-quality performance than the traditional Boolean approach. According to the definition modeled in Eq (22). The values of ones in binary solution represent the features that should be selected features while removed those that corresponding to zero values.

**Algorithm 1. Proposed MRFODE feature selection method.**

1. Input: Extracted features from COVID-19 x-ray images

2. Set the initial value for the parameters of MRFODE.

3. Split features into two training and testing sets

4. Generate a set of *N* agents (*X*).

5. Using Eq (23) to compute the fitness function of *x*_*i*_ based on the training set.

6. Find the best solution *x*_*best*_.

7. While (terminal condition not reached).

8. Using Eq (22) to convert each *x* to binary.

9. Using Eq (23) to compute the fitness function of each *x*

10.     Find the best solution *x*_*best*_

11.         For *i* = 1:*N*

12.         If *rand*<0.5

13.             If *t*/*T*<*rand*

14.                 Using Eq (14) to update *x*_*i*_

15.             Else

16.               Using Eq (16) to update *x*_*i*_

17.               END IF **Cyclone foraging**

18.             Else

19.                 Using Eq (11) to update *x*_*i*_

          20. END IF

21.       END-FOR

22.         For *i* = 1:*N*

23.         Compute the probability using Eq (24)

24.         If *Pr*_*i*_<0.5

25.         Update *x*_*i*_ using MRFO.

26.     ELSE

27.         Update *x*_*i*_ using DE.

28.     END-FOR

29.         *t* = *t*+1

30.     End While

31.     Reduce the testing set according to *x*_*best*_, and using KNN to predict the target.

32.     Evaluate the quality of the model.

To illustrate this concept, consider the value of the current agent in binary form is *x*_*i*_ = [1,0,0,1,1], so this indicates that the second and the third features will remove while others selected as relevant features. The process of converting the real solution to Boolean is followed by computing the quality of the selected features using the following equation:

$$f_{i}=\beta \times \gamma +\left(1-\beta \right)\times \frac{N_{sel}}{D},$$ (23)

In Eq (23), *γ* refers to the classification error by using the KNN classifier. The *N*_*sel*_ represents the number of features selected by the current agent. The *β*∈[0,1] is a random value applied to provides a balance between *γ* and the selected features. Then the best agent (*x*_*best*_) found in our study, which has the smallest. Then the agents are updated according to the operators of MRFO algorithm or DE, as discussed in Sections C .1 and C. 2, respectively. This process performed by computing the probability (*Pr*_*i*_) of each agent in Somersault foraging as in Eq 24.

$$Pr_{i}=\frac{f_{i}}{\sum_{i=1}^{N}f_{i}}$$ (24)

In the case of *Pr*_*i*_<0.5 then the operators of MRFO are used to update *x*_*i*_; otherwise, the operators of DE used. After reaching the terminal conditions the best agent (*x*_*best*_) is a return from this second phase.

In the third phase, the testing set applied to assess the selected features from the second phase, which performed by removing the irrelevant features—followed by evaluating the performance of classification using a variant set of metrics.

### 2.4. The proposed model summary

**Fig 2:**
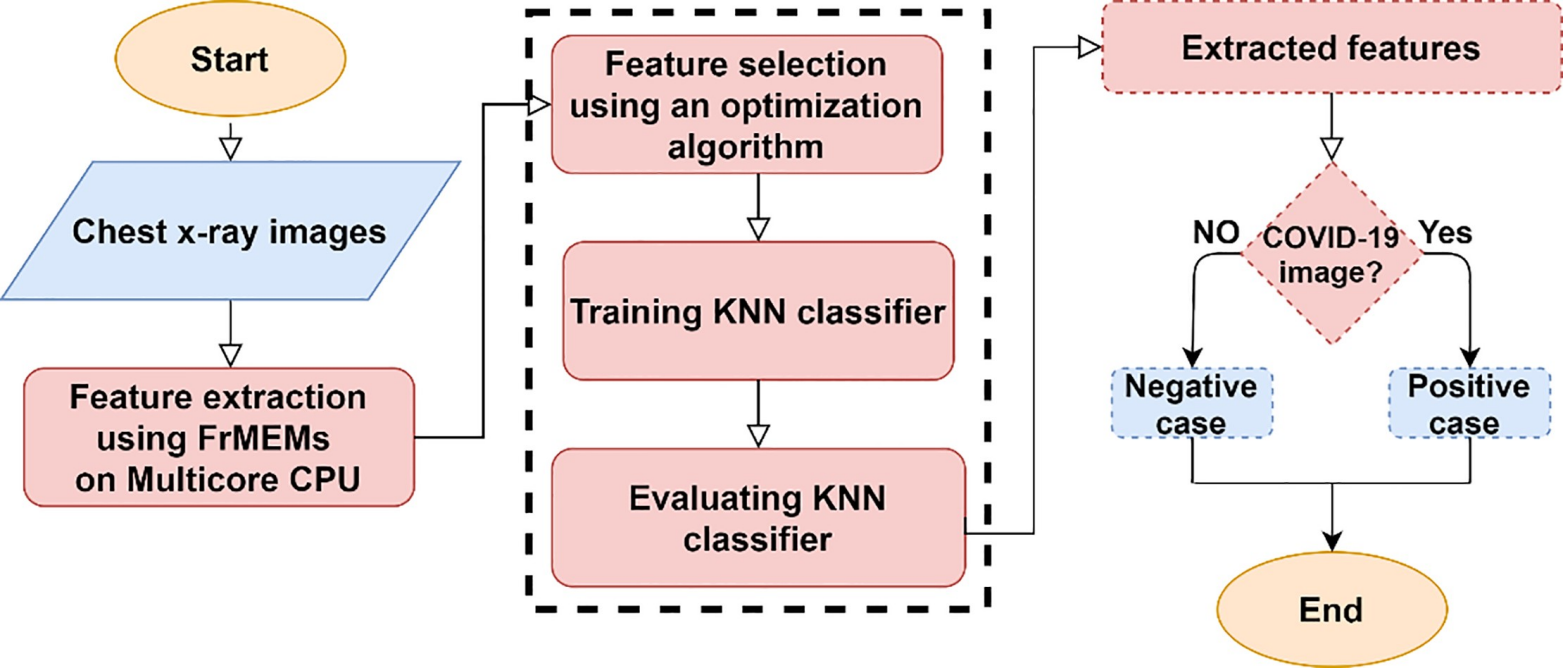
Flowchart of the proposed method.

Fig 2 depicts the flowchart of the proposed classification method of chest x-ray images which summarizes the entire model components. The input to the classifier is a set of images of two classes, COVID-19, and normal cases. The parallel FrMEMs is executed on multi-core CPUs to extract the image features. Then, an optimization algorithm used for the purposed of feature extraction. Finally, a KNN classifier trained and evaluated.

## 3. Experiments

### 3.1. Datasets

We used two different datasets for this study. The first dataset collected by Joseph Paul Cohen and Paul Morrison and Lan Dao in GitHub [31] and images extracted from 43 different publications. References of each image provided in the metadata. Normal and Viral pneumonia images adopted from the chest x-ray Images (pneumonia) database [32]. It contains 216 COVID-19 positive images (some collected from the Twitter account of Italian Cardiothoracic radiologist), 1,675 negative COVID-19 images. The data was collected mainly from retrospective cohorts of pediatric patients of one to five years old from Guangzhou Women and Children's medical center. We refer to this dataset as dataset-1.

While the other dataset collected by a team of researchers from Qatar University, Doha, Qatar, and the University of Dhaka, Bangladesh, along with their collaborators from Pakistan and Malaysia in collaboration with medical doctors [33]. In addition to [31] and [32], they have added images from the Italian Society of Medical and Interventional Radiology (SIRM) COVID-19 DATABASE [34]. This dataset consists of 219 COVID-19 positive images and 1,341 negative COVID-19 images. We refer to this dataset as dataset-2.

**Fig 3:**
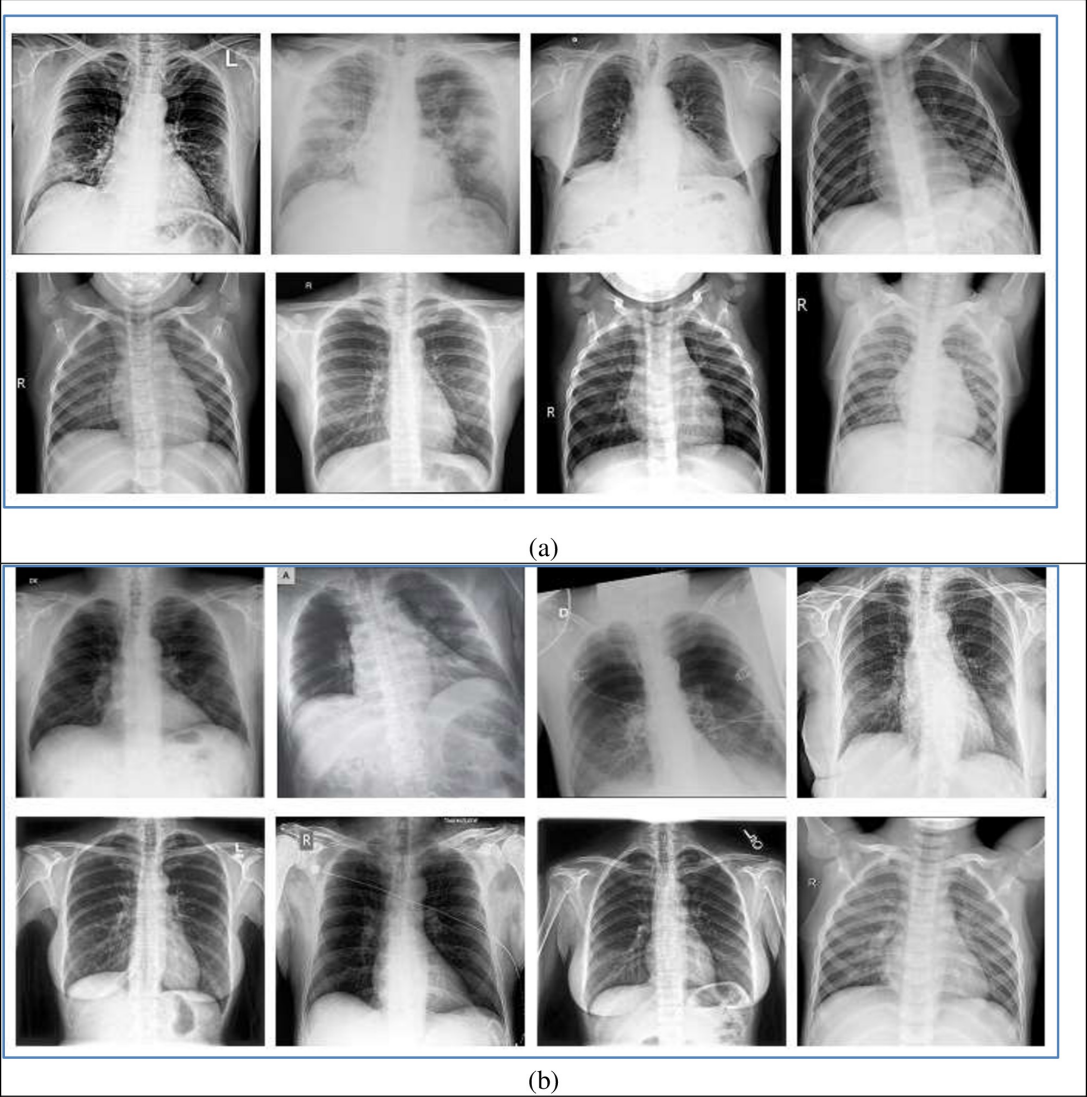
(A) Sample images of dataset-1 (B) Sample images of dataset-2.

Both datasets shared many characteristics regarding the collecting source. For both datasets, the COVID-19 images collected from a patient with an age range from 40 to 84 from both genders. The data contains 216 COVID-19 positive images and 1,675 COVID-19 negative images. Sample images of both datasets shown in Fig 3.

### 3.2. Evaluation of the proposed model

In this study, the results of the proposed COVID-19 x-ray classification image-based method compared with other popular MH techniques that applied as FS. These techniques include sine cosine algorithm (SCA), grey wolf optimization (GWO), Henry Gas Solubility optimization (HGSO), whale optimization algorithm (WOA), and Harris Hawks optimizer (HHO).

These algorithms are used in this comparison since they established their performance in different applications such as global optimization and feature selection methods [35–39]. The quality of each FS algorithm assessed by using three measures: Accuracy, the ratio of the selected features, and the fitness value where the accuracy (Acc) defined as:

$$Acc=\frac{T_{P}+T_{N}}{T_{N}+T_{P}+F_{N}+F_{P}}\times 100$$ (25)

### *3*.*3*. Results and discussion

In this subsection, we described the performed experiments and discussed the obtained results. Table 1 lists the run-time in seconds and the obtained speedup of the moment computation, i.e., feature extraction phase, at moment order equals and 30 to extract 961 features from each image. The obtained speedup is close to the theoretical limits (2x, 4x & 8x for 2-, 4- and 8-multi-core), which prove the efficiency of the utilized parallel approach. The results of Table 1 show that the proposed parallel implementation of the moment computation accelerating the feature extraction phase by a factor related to the number of used CPU cores.

**Table 1:** The running time in seconds required to extract 961 features from one image.

	1 core (sequential)	2 cores	4 cores	8 cores
Run-time	213	118	55.47	27.1
speedup	--	1.80×	3.83×	7.84×

The proposed algorithm depends on extracting the features using FrMEMs and using a modified MRFO based on DE as a feature selection method. To find the smallest subset of relevant features that leads to increase the classification performance. Besides, the MRFODE compared with other MH methods that used as feature selection models, including such as MRFO, HGSO, HHO, GWO, SCA, and WOA. These FS methods are used the extracted features from FrMEMs as input and aimed to select the most relevant features. The Comparison results according to accuracy, several selected features, and fitness value given in Tables 2 and 3.

**Table 2:** Comparison results of MRFODE and other MH methods in terms of accuracy.

Fn	Measure	MRFO	MRFODE	HHO	HGSO	WOA	SCA	GWO
Dataset-1	Mean	0.9499	0.9609	0.9414	0.9456	0.9541	0.9536	0.9551
	STD	0.0081	0.0106	0.0136	0.0076	0.0048	0.0048	0.0079
	*N*_*Sel*_	15.6	16	20	16.8	22	97.2	105.2
	*RSF*	0.015	0.0166	0.02	0.017	0.022	0.1	0.109
Dataset-2	Mean	0.9688	0.9809	0.9274	0.9452	0.9490	0.9618	0.9637
	STD	0.0097	0.0135	0.0328	0.0122	0.0164	0.0060	0.0094
	*N*_*Sel*_	25.6	18.8	31.6	20.1	21	91.4	107.4
	*RSF*	0.0266	0.019	0.032	0.0208	0.022	0.094	0.0111

**Table 3:** Results of fitness value for MRFODE and other methods.

		MRFO	MRFODE	HHO	HGSO	WOA	SCA	GWO
**Dataset-1**	Mean	0.0332	**0.0278**	0.0355	0.0359	0.0344	0.1734	0.1292
	STD	**0.0030**	0.0072	0.0071	0.0070	0.0034	0.0648	0.0046
	Best	0.0299	**0.0189**	0.0284	0.0266	0.0305	0.1291	0.1252
	Worst	**0.0365**	0.0386	0.0437	0.0426	0.0380	0.3516	0.1362
**Dataset-2**	Mean	0.0289	**0.0257**	0.0423	0.0362	0.0364	0.1615	0.1261
	STD	0.0045	**0.0026**	0.0156	0.0066	0.0080	0.0642	0.0064
	Best	0.0231	**0.0215**	0.0296	0.0311	0.0254	0.1164	0.1184
	Worst	0.0337	**0.0280**	0.0594	0.0470	0.0460	0.3363	0.1342

It observed from Table 2 that the MRFODE provides better accuracy than other MH methods based on the Best and mean of the accuracy among the two datasets. Since it achieves the first rank in both terms, followed by GWO that has the second rank. Meanwhile, the SCA algorithm is ranked #1 in terms of STD followed by HGSO and GWO at dataset-1 and dataset-2, respectively. As well as, the accuracy of using the extracted features without the feature selection method is the proposed model 0.901 and 0.9309 for Dataset-1 and Dataset-2, respectively. These results indicate the high effect of proposed MRFODE on the quality of classification the COVID-19 x-ray images.

In terms of the fitness value, it is seen from Table 3 that the proposed MRFODE has the smallest fitness value overall the mean, STD, Best, and Worst values at Qatar dataset. However, at the data1, it provides better results according to the mean and the Best value, which is ranked 1#, while, the traditional MRFO achieves the better at STD, and Worst. These results indicate that the proposed algorithm has a high ability to balance between the error of classification through selected the most relevant features, as well as, and, selecting the smallest number of features.

Moreover, Table 2 lists the average of MRFODE and other MH methods in terms of several selected features. It noticed that the proposed MRFODE picks the smallest number of features at the two datasets.

**Fig 4:**
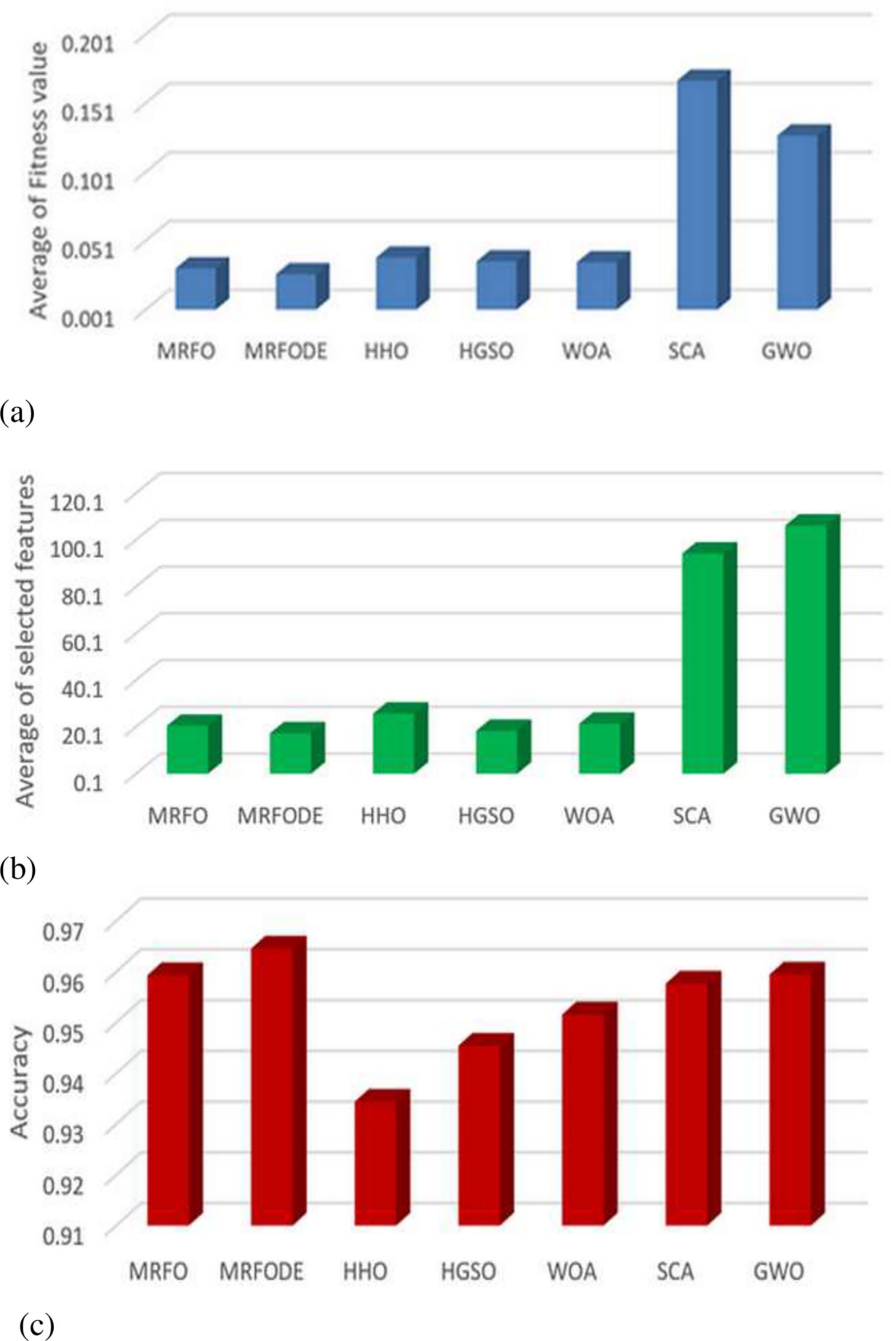
Average of comparison results between algorithm over (a) accuracy, (b) a number of selected features, and (c) fitness value.

Fig 4 depicts the average of MRFODE and other MH methods overall the two datasets according to the accuracy, number of selected features, and fitness value. The results shown in Fig 4 provides evidence for the superiority of the proposed MRFODE since it has a high value at accuracy. Also, the smallest number of selected features and fitness value. However, the CPU time(s) of it is the third rank, and this the main limitation of it.

**Fig 5:**
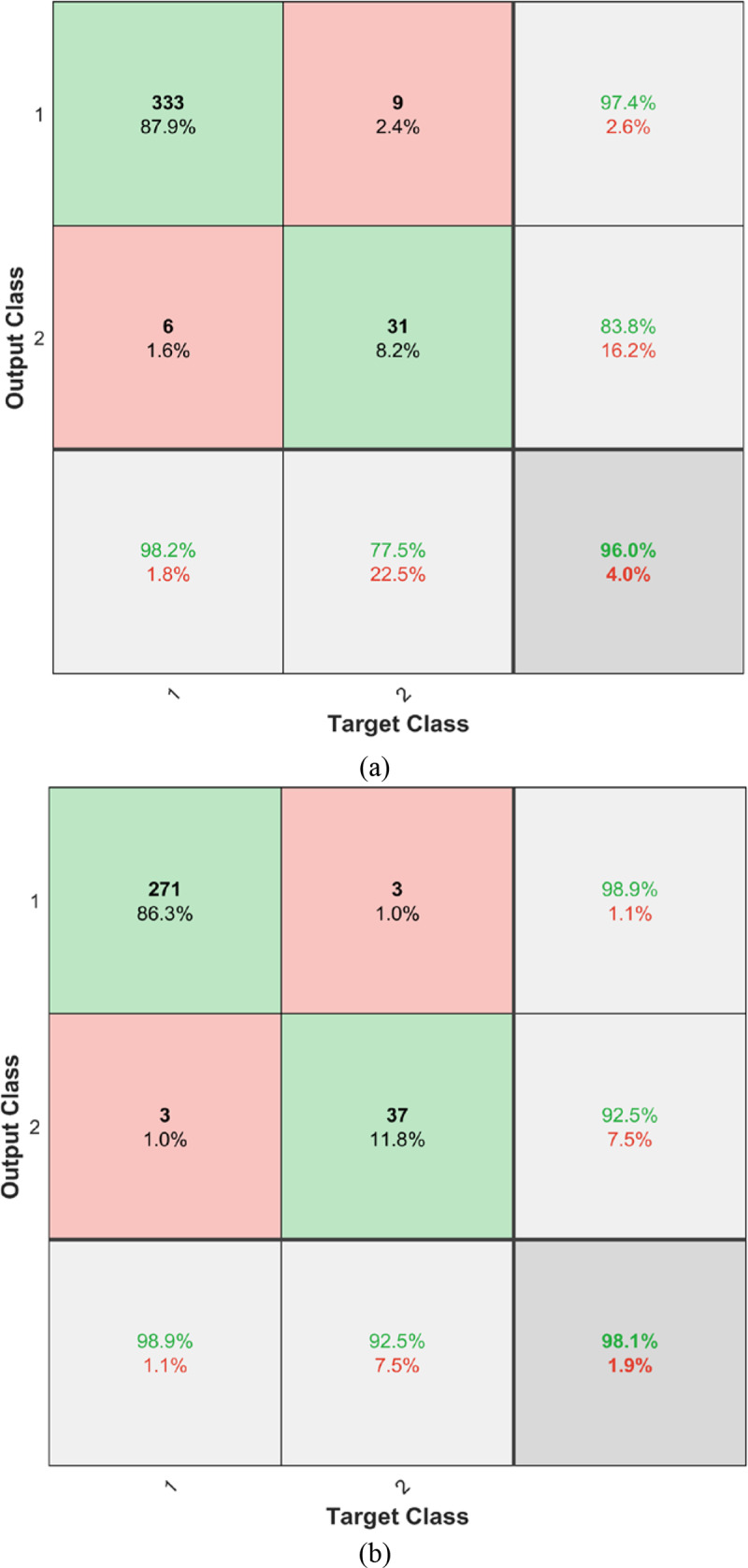
Confusion matrix using MRFODE for (A) dataset-1 and (B) dataset-2.

Fig 5 depicts the confusion matrix for the two datasets using the predicted output from MRFODE. From Fig 5, it can notice the high ability of the proposed model to distinguish the COVID-19 from non-COVID x-ray images.

The further analysis presented to evaluate the performance of the proposed model by using a non-parametric test named Friedman test, which ranks the methods. For the accuracy measure, the best algorithm is that it has the highest rank, while for the other measures, the lowest rank preferred.

Table 4 lists the mean rank of each algorithm obtained using the Friedman test. From these results, it noticed that the developed MRFODE has the best rank at the accuracy, selected features, and fitness value. Since it has a higher rank at accuracy and the smallest mean rank at the other two measures. This indicates the high ability of MRFODE to select the optimal subset of features that leads to an increase in the classification accuracy for the two tested datasets.

**Table 4:** Mean rank obtained using Friedman test for each method.

	MRFO	MRFODE	HHO	HGSO	WOA	SCA	GWO
Accuracy	5.3750	6.6250	1.1250	1.8750	3.6250	3.8750	5.5000
Fitness	2	1.8750	4.6250	3.8750	3.3750	6.8750	5.3750
Attribute	2.5000	1.5000	4.5000	2.5000	4	6	7

### 3.4. Comparison with deep neural networks

In this subsection, the performance of the proposed approach compared to other convolutional neural networks. In [12], the proposed convolutional neural network (CNN) model for image classification surpasses the reported human-level performance.

While CNN achieves the best results on large data sets, they require a lot of data and computational resources to train. In many cases, the dataset is limited and may not be sufficient to train a CNN from scratch. In such a scenario, to leverage the power of CNNs and, at the same time, reduce the computational costs, transfer learning can be used [40, 41]. In this approach, the network trained using a large and diverse generic image data set and then applied to a specific task [42]. There are several pre-trained neural networks have won international competitions like VGGNet [12], Resnet [43], Nasnet [44], Mobilenet [45], Inception (GoogLeNet) [46] and Xception [47]. In this paper, we compare our model with MobileNet due to resource limitations. Table 4 presents a comparison with Mobilenet and related works on both datasets.

In Table 5, the proposed approach achieved high accuracy among other deep neural networks (DNN) and compared it to the only available published paper in this dataset. It turns out that the proposed approach, which has only 16 and 18 features for both dataset-1 and dataset-2, respectively, achieves better results in most classification criteria than one of the most popular DNN structures with a feature set which has about 50K features. The proposed approach achieves both high performances with the least number of features, which implies better resource consumption and time-saving.

**Table 5:** Comparison with MobileNet and related works.

Model	Number of features	Performance			Dataset
		Accuracy	Recall	Precision	
Apostolopoulos [13]	--	0.9678	0.9866	0.9646	Dataset 1
MobileNet	50176	0.9603	0.9665	0.9884	
**Proposed**	**16**	**0.9609**	**0.9875**	**0.9875**	
MobileNet	50176	0.9967	0.9992	0.9970	Dataset 2
Chowdhury [30]	--	98.3	100	96.7	
**Proposed**	**18.8**	**0.9809**	**0.9891**	**0.9891**	

## 4. Conclusion

In this study, we proposed a method for the visual diagnosis of COVID-19 cases on chest x-ray images. The proposed utilized a fractional moment (i.e., FrMEMs) to extract features of the COVID-19 x-ray images. Then, a modified version from Manta Ray Foraging Optimization (MRFO) applied as a feature selection method, which modified using DE to improve the ability of MRFO to find the relevant features from those extracted features. In the proposed MRFODE feature selection method, the KNN classifier utilized to decide whether a given chest x-ray image as a COVID-19 or normal case. The proposed method evaluated on two different datasets. Comparing to a successful CNN architecture, the MobileNet model, the proposed method achieved comparable performance on the accuracy, recall, and precision evaluation metrics with the least number of features. The proposed approach achieved both high performances as well as resource consumption by selecting the most significant features. Our future work might include other applications from the medical and other relevant fields.

## Supporting information

S1 Appendix Table of abbreviations. (DOCX)

## References

[1] Hosseini M. S. and Zekri M., "Review of medical image classification using the adaptive neuro-fuzzy inference system," *Journal of medical signals and sensors*, vol. 2, p. 49, 2012 23493054 PMC3592505

[2] Quek C., Irawan W., and Ng E., "A novel brain-inspired neural cognitive approach to SARS thermal image analysis," *Expert Systems with Applications*, vol. 37, pp. 3040–3054, 2010.

[3] Xie X., Li X., Wan S., and Gong Y., "Mining x-ray images of SARS patients," in *Data Mining*, 2006, pp. 282–294.

[4] Rajpurkar P., Irvin J., Zhu K., Yang B., Mehta H., Duan T., et al, "Chexnet: Radiologist-level pneumonia detection on chest x-rays with deep learning," *arXiv preprint ar X iv*:1711 05225, 2017.

[5] Ke Q., Zhang J., Wei W., Połap D., Woźniak M., Kośmider L., et al, "A neuro-heuristic approach for recognition of lung diseases from X-ray images," *Expert Systems with Applications*, vol. 126, pp. 218–232, 2019.

[6] Chouhan V., Singh S. K., Khamparia A., Gupta D., Tiwari P., Moreira C., et al, "A Novel Transfer Learning-Based Approach for Pneumonia Detection in Chest X-ray Images," *Applied Sciences*, vol. 10, p. 559, 2020.

[7] Rawat W. and Wang Z., "Deep convolutional neural networks for image classification: A comprehensive review," *Neural computation*, vol. 29, pp. 2352–2449, 2017 10.1162/NECO_a_00990 28599112

[8] L. Yang, Y. Zhang, J. Chen, S. Zhang, and D. Z. Chen, "Suggestive annotation: A deep active learning framework for biomedical image segmentation," in *International conference on medical image computing and computer-assisted intervention*, 2017, pp. 399–407.

[9] Y. Yang and S. Newsam, "Comparing SIFT descriptors and Gabor texture features for classification of remote sensed imagery," in *2008 15th IEEE international conference on image processing*, 2008, pp. 1852–1855.

[10] Elaziz M. A., Hosny K. M., and Selim I., "Galaxies image classification using artificial bee colony based on orthogonal Gegenbauer moments," *Soft Computing*, vol. 23, pp. 9573–9583, 2019.

[11] Suykens J. A. and Vandewalle J., "Least squares support vector machine classifiers," *Neural processing letters*, vol. 9, pp. 293–300, 1999.

[12] Simonyan K. and Zisserman A., "Very deep convolutional networks for large-scale image recognition," *arXiv preprint ar X iv*:1409 1556, 2014.

[13] Apostolopoulos I. D. and Mpesiana T. A., "Covid-19: automatic detection from x-ray images utilizing transfer learning with convolutional neural networks," *Physical and Engineering Sciences in Medicine*, p. 1, 2020. 10.1007/s13246-020-00865-4 PMC7118364 32524445

[14] Narin A., Kaya C., and Pamuk Z., "Automatic detection of coronavirus disease (COVID-19) using X-ray images and deep convolutional neural networks," *arXiv preprint ar X iv*:2003 10849, 2020. 10.1007/s10044-021-00984-y PMC8106971 33994847

[15] Hosny K. M., Hamza H. M., and Lashin N. A., "Copy-for-duplication forgery detection in colour images using QPCETMs and sub-image approach," *IET Image Processing*, vol. 13, pp. 1437–1446, 2019.

[16] Eltoukhy M. M., Elhoseny M., Hosny K. M., and Singh A. K., "Computer aided detection of mammographic mass using exact Gaussian–Hermite moments," *Journal of Ambient Intelligence and Humanized Computing*, pp. 1–9, 2018.

[17] Hosny K., Elaziz M., Selim I., and Darwish M., "Classification of galaxy color images using quaternion polar complex exponential transform and binary Stochastic Fractal Search," *Astronomy and Computing*, p. 100383, 2020.

[18] Flusser J., Suk T., and Zitová B., *2D*, *and 3D image analysis by moments*: John Wiley & Sons, 2016.

[19] Hu H.-t., Zhang Y.-d., Shao C., and Ju Q., "Orthogonal moments based on exponent functions: Exponent-Fourier moments," *Pattern Recognition*, vol. 47, pp. 2596–2606, 2014.

[20] Hosny K. M. and Darwish M. M., "New set of multi-channel orthogonal moments for color image representation and recognition," *Pattern Recognition*, vol. 88, pp. 153–173, 2019.

[21] Singh C. and Singh J., "Multi-channel versus quaternion orthogonal rotation invariant moments for color image representation," *Digital Signal Processing*, vol. 78, pp. 376–392, 2018.

[22] Wang X.-y., Li W.-y., Yang H.-y., Wang P., and Li Y.-w., "Quaternion polar complex exponential transform for invariant color image description," *Applied Mathematics and Computation*, vol. 256, pp. 951–967, 2015.

[23] T. Suk and J. Flusser, "Affine moment invariants of color images," in International Conference on Computer Analysis of Images and Patterns, 2009, pp. 334–341.

[24] Hosny K. M. and Darwish M. M., "New set of quaternion moments for color images representation and recognition," *Journal of Mathematical Imaging and Vision*, vol. 60, pp. 717–736, 2018.

[25] Hosny K. M. and Darwish M. M., "A Kernel-Based method for Fast and accurate computation of PHT in polar coordinates," *Journal of Real-Time Image Processing*, vol. 16, pp. 1235–1247, 2019.

[26] Qin H., Qin L., Xue L., and Yu C., "A parallel recurrence method for the fast computation of Zernike moments," *Applied Mathematics and Computation*, vol. 219, pp. 1549–1561, 2012.

[27] A.-W. Deng, C.-H. Wei and C.-Y. Gwo, "Algorithms for computing Zernike moments and image reconstruction in parallel process," in *2015 2nd International Conference on Information Science and Control Engineering*, 2015, pp. 105–109.

[28] Salah A., Li K., Hosny K. M., Darwish M. M., and Tian Q., "Accelerated CPU–GPUs implementations for quaternion polar harmonic transform of color images," *Future Generation Computer Systems*, vol. 107, pp. 368–382, 2020.

[29] Zhao W., Zhang Z., and Wang L., "Manta ray foraging optimization: An effective bio-inspired optimizer for engineering applications," *Engineering Applications of Artificial Intelligence*, vol. 87, p. 103300, 2020.

[30] Qin A. K., Huang V. L., and Suganthan P. N., "Differential evolution algorithm with strategy adaptation for global numerical optimization," *IEEE transactions on Evolutionary Computation*, vol. 13, pp. 398–417, 2008.

[31] Cohen J. P., Morrison P., and Dao L., "COVID-19 image data collection," *arXiv preprint ar X iv*:2003 11597, 2020.

[32] P. Mooney. (2020, 2020-April-11). Chest X-Ray Images (Pneumonia). Available: https://www.kaggle.com/paultimothymooney/chest-xray-pneumonia

[33] Chowdhury M. E., Rahman T., Khandakar A., Mazhar R., Kadir M. A., Mahbub Z. B. et al, "Can AI help in screening Viral and COVID-19 pneumonia? " *arXiv preprint ar X iv*:2003 13145, 2020.

[34] D. A. L. Izzo Andrea. (2020, April-11-2020). Radiology. (2020). COVID-19 Database. Available: https://www.sirm.org/category/senza-categoria/covid-19/

[35] M. E. A. Elaziz, A. A. Ewees, D. Oliva, P. Duan, and S. Xiong, "A hybrid method of sine cosine algorithm and differential evolution for feature selection," in International Conference on Neural Information Processing, 2017, pp. 145–155.

[36] Ibrahim R. A., Elaziz M. A., and Lu S., "Chaotic opposition-based grey-wolf optimization algorithm based on differential evolution and disruption operator for global optimization," *Expert Systems with Applications*, vol. 108, pp. 1–27, 2018.

[37] Ewees A. A., and Hassanien A. E., "Multi-objective whale optimization algorithm for content-based image retrieval," *Multimedia Tools and Applications*, *77*, *pages* 26135–26172, 2018.

[38] Hashim F. A., Houssein E. H., Mabrouk M. S., Al-Atabany W., and Mirjalili S., "Henry gas solubility optimization: A novel physics-based algorithm," *Future Generation Computer Systems*, vol. 101, pp. 646–667, 2019.

[39] Hans R., Kaur H., and Kaur N., "Opposition-based Harris Hawks optimization algorithm for feature selection in breast mass classification," *Journal of Interdisciplinary Mathematics*, vol. 23, pp. 97–106, 2020.

[40] A. Sharif Razavian, H. Azizpour, J. Sullivan, and S. Carlsson, "CNN features off-the-shelf: an astounding baseline for recognition," in Proceedings of the IEEE conference on computer vision and pattern recognition workshops, 2014, pp. 806–813.

[41] J. Donahue, Y. Jia, O. Vinyals, J. Hoffman, N. Zhang, E. Tzeng, et al., "Decaf: A deep convolutional activation feature for generic visual recognition," in International conference on machine learning, 2014, pp. 647–655.

[42] L. D. Nguyen, D. Lin, Z. Lin, and J. Cao, "Deep CNNs for microscopic image classification by exploiting transfer learning and feature concatenation," in 2018 IEEE International Symposium on Circuits and Systems (ISCAS), 2018, pp. 1–5.

[43] K. He, X. Zhang, S. Ren, and J. Sun, "Deep residual learning for image recognition," in Proceedings of the IEEE conference on computer vision and pattern recognition, 2016, pp. 770–778.

[44] Zoph B., Vasudevan V., Shlens J., and Le Q., "AutoML for large scale image classification and object detection," *Google AI Blog*, vol. 2, p. 2017, 2017.

[45] Howard A. G., Zhu M., Chen B., Kalenichenko D., Wang W., Weyand T., et al, "Mobilenets: Efficient convolutional neural networks for mobile vision applications," *arXiv preprint ar X iv*:1704 04861, 2017.

[46] C. Szegedy, W. Liu, Y. Jia, P. Sermanet, S. Reed, D. Anguelov, et al., "Going deeper with convolutions," in Proceedings of the IEEE conference on computer vision and pattern recognition, 2015, pp. 1–9.

[47] F. Chollet, "Xception: Deep learning with depthwise separable convolutions," in Proceedings of the IEEE conference on computer vision and pattern recognition, 2017, pp. 1251–1258.

