# Supplementary material for: New machine learning method for image-based diagnosis of COVID-19
Source: PLoS One. 2020 Jun 26;15(6):e0235187. doi: 10.1371/journal.pone.0235187 (PMC7319603; doi:10.1371/journal.pone.0235187)
Supplement: S1 Appendix — (DOCX) [file pone.0235187.s001.docx]

Appendix: Table of Abbreviations

**Table A:** List of Abbreviations

| **Abbreviations** | **Description** |
| --- | --- |
| WHO | World Health Organization |
| FrMEMs | Fractional Multi-channel Exponent Moments |
| SARS | Severe Acute Respiratory Syndrome |
| ML | Machine Learning |
| CNN | Convolutional Neural Networks |
| MRFODE | Manta-ray Foraging Optimization and Differential Evolution |
| MRFO | Manta-ray Foraging Optimization |
| DE | Differential Evolution |
| SIRM | Italian Society of Medical and Interventional Radiology |
| SCA | Sine Cosine Algorithm |
| GWO | Grey Wolf Optimization |
| HGSO | Henry Gas Solubility Optimization |
| WOA | Whale Optimization Algorithm |
| HHO | Harris Hawks Optimizer |
